# The effect of psychosocial factors on breast cancer outcome: a systematic review

**DOI:** 10.1186/bcr1744

**Published:** 2007-07-17

**Authors:** Matthew E Falagas, Effie A Zarkadoulia, Eleni N Ioannidou, George Peppas, Christos Christodoulou, Petros I Rafailidis

**Affiliations:** 1 Alfa Institute of Biomedical Sciences (AIBS), Athens, Greece; 2 Department of Medicine, Tufts University School of Medicine, Boston, Massachusetts, USA; 3 Department of Medicine, Henry Dunant Hospital, Athens, Greece; 4 Department of Surgery, Henry Dunant Hospital, Athens, Greece; 5 Department of Medical Oncology, Henry Dunant Hospital, Athens, Greece

## Abstract

**Introduction:**

We sought to review the available evidence regarding the effect of psychosocial factors on the survival of breast cancer patients.

**Methods:**

We systematically searched the PubMed and PsycINFO databases to identify relevant studies.

**Results:**

We identified 31 studies examining the association of various psychosocial parameters with overall breast cancer survival/disease free survival and 6 studies examining whether psychological intervention influences the disease outcome. Of the 31 studies summarized in this overview, 25 (80.6%) showed a statistically significant association between at least one psychosocial variable and disease outcome. Parameters associated with better breast cancer prognosis are social support, marriage, and minimizing and denial, while depression and constraint of emotions are associated with decreased breast cancer survival; however, the role of these factors has not been verified in all studies.

**Conclusion:**

Most of the studies show a significant relationship between psychosocial factors and survival, but the actual psychosocial variables related to survival are not consistently measured across studies and the findings for many of the psychosocial variables with survival/recurrence are not consistent across studies. Thus, more research is warranted regarding the role of social support, marriage, minimizing and denial, depression and constraint of emotions on breast cancer survival.

## Introduction

Breast cancer is a significant cause of morbidity and mortality among women; it is the second leading cause of death due to cancer in women, exceeded only by lung cancer (American Cancer Society 2007) [1]. The fact that patients with apparently similar tumours at the time of presentation differ significantly in the time of relapse and overall survival implies that the determinants of survival could be broader than initially assumed in the purely medical framework. The question of whether psychosocial parameters could have an effect on the clinical outcome of cancer in general has yielded a large body of research devoted to this issue and has divided the medical community into believers and non-believers. We reviewed articles on the psychosocial correlates of cancer relapse and survival as well as on the impact of psychological intervention on survival from 1979 to 2006.

## Materials and methods

### Literature search

We performed a systematic search of the literature to obtain original studies that investigated possible associations between psychosocial/psychological factors and survival among patients with breast cancer. The relevant studies were identified by the use of the PubMed database [2]. In our primary search, conducted on 16 to 17 May 2006, we used two different strategies with the following key words: first, breast cancer AND (prognosis OR outcome) AND (psychological OR psychosocial OR cognitive) factors; and second, breast cancer AND (prognosis OR outcome) AND personality. Afterwards, we attempted additional searches using various combinations that included the following key words as well: positive outcome, optimism, breast cancer diagnosis, social support, decreased mortality, confounding variables, patient autonomy, patient support, disease recovery, social networks, quality of life, beneficial effect, survivors. Finally, we extended our search by looking into the PsychINFO database to find relevant studies [3].

### Study selection

Our systematic review included original studies written in the English language; thus, reviews, editorials, letters, and case reports were excluded from our study. Studies included in our review contained quantitative data regarding the association of psychological/psychosocial factors with breast cancer disease outcomes (survival and recurrence). Moreover, we included studies that examined the impact of psychological intervention programs on patient survival. We did not use any limitations regarding study sample size, study design, and specific measures of outcomes used in the various studies. We excluded studies that focused on the impact of breast surgery and adjuvant therapy on patients' quality of life, psychological and psychosocial well-being, and survival. Studies referring solely to the influence of biological and organic factors on survival were not included in our review. We also left out studies that concentrated on the impact of psychological, psychosocial, or cognitive factors on the onset of the disease. Finally, we did not include studies that focused on the evaluation of intervention programs to enhance self-management and coping with illness.

### Data extraction

From the studies that were included in our review we extracted data regarding the date of publication, the type of the study, the setting of the study, the study population, the aim of the study, the follow-up period, the method used to assess and measure psychological and psychosocial factors, the disease outcome (that is, survival/disease free survival and recurrence), the specific psychological/psychosocial factors examined as potentially influencing breast cancer survival or prognosis, and the main findings regarding statistically significant associations between those factors and breast cancer prognosis/outcome.

## Results

In total we identified 37 studies examining psychosocial factors and breast cancer survival; 31 descriptive analytical studies and 6 studies with psychological intervention. In Tables 1 and 2 we summarize the 31 studies regarding psychosocial/psychological factors and survival or recurrence among patients with breast cancer [4-34]. In Tables 3 and 4 we summarize the six studies regarding the impact of psychosocial intervention on survival of breast cancer patients [35-40]. In Table 5 we summarize the association of psychosocial factors with survival/recurrence. A total number of 61,611 female patients with breast cancer were examined regarding psychosocial factors and survival (52,857 of them in retrospective studies). Of the total 31 identified descriptive analytical studies, 15 were conducted in the US [5-8,10,11,15,16,22,24,26,27,31,32,34], 2 in Canada [9,29], 1 in Israel [18], 2 in Australia [23,30], 1 in Japan [19] and the rest of them in Europe (6 in the UK, [4,12-14,21,25] 1 in Switzerland and Germany [17], 1 in Denmark [28], 1 in Belgium [20] and 1 in Finland [33]). Two studies examined the same cohort of women to assess the impact of psychosocial factors on survival [26] and the effect of cancer specific beliefs on survival [27]. Out of the 31 studies that did not employ psychological intervention, 12 examined patients with all stages of breast cancer [6,7,9,12,15,19,22,26-28,32,34]. Five and two studies examined patients with metastatic/recurrent cancer [5,8,11,23,24] and invasive stage I or II to III cancer [20,31], respectively, whereas twelve studies examined patients with early stage cancer [4,10,13,14,16-18,21,25,29,30,33] characterized either as operable, stage I and II in the TNM classification (that is, the staging system based on tumour size, lymph node involvement, and the presence of metastasis), or localized regional and non-metastatic. Fourteen studies focused on patients' reactions to having cancer and their feelings whereas nine focused on social factors (that is, marital status, social ties, religion) that can offer significant support to breast cancer patients, and seven studies focused on both. One study examines the effect of beliefs about cancer curability.

**Table 1:** Characteristics of studies examining psychosocial factors associated with breast cancer survival

Reference	Setting	Type of study	Study population	Aim of the study
Greer *et al*. 1979 [4]	King's College Hospital, UK	Prospective	69 women with early breast cancer (T 0–1, N 0–1, M 0)	Examination of the effect of the psychological response to breast cancer on outcome
Derogatis *et al*. 1979 [5]	Johns Hopkins Oncology Center, Baltimore, USA	Prospective	35 women under treatment for metastatic breast cancer	Examination of the effect of psychological factors on breast cancer survival
Marshall *et al*. 1983 [6]	Roswell Park Memorial Institute	Cohort analysis	352 white women with primary breast cancer presenting between 1958 and 1960	Examination of the effect of the social environment on breast cancer survival
Jensen *et al*. 1987 [7]	USA	Prospective	Three groups (G) of women: G1 (*n *= 25), breast cancer recurrent or metastatic; G2 (*n *= 27), breast cancer without recurrence; G3 (*n *= 34), no history of cancer	Examination of the effect of psychological factors on the survival/recurrence
Jamison *et al*. 1987 [8]	Adult outpatient Oncology Clinic of Vanderbilt University Hospital, Nashville	Prospective	49 women with metastatic breast cancer	Examination of the effect of psychosocial factors on breast cancer survival
Hislop *et al*. 1987 [9]	A Maxwell Evans Clinic, Vancouver, Canada	Prospective	133 women with primary ductal breast cancer (stages I to IV) diagnosed between June 1980 and May 1981	Examination of the effect of psychosocial factors on breast cancer survival/recurrence
Cassileth *et al*. 1988 [10]	University of Pennsylvania Cancer Center, USA	Prospective (controlled)	204 patients with advanced, prognostically poor malignant disease at diagnosis (Group I), and 155 patients with intermediate or high-risk melanoma or stage II breast cancer (Group II); breast cancer patients *n *= 88	Examination of the effect of psychosocial factors on breast cancer survival in an 8 year follow-up period
Levy *et al*. 1988 [11]	NIH Clinical Center	Prospective	36 women with recurrent breast cancer	Examination of the effect of biological and psychological factors on breast cancer survival
Ramirez *et al*. 1989 [12]	Guy's hospital, London Lewisham Hospital, London	Case control	50 women who had developed a first recurrence after treatment for operable breast cancer, 50 who had operable breast cancer in remission	Examination of the effect of stressful life events on breast cancer recurrence
Barraclough *et al*. 1992 [13]	Breast clinics in Southampton and Portsmouth	Prospective	204 breast cancer patients without clinical evidence of spread beyond the axilla, treated either with mastectomy or wide excision followed by radiotherapy	Examination of the effect of psychosocial factors on breast cancer recurrence
Morris *et al*. 1992 [14]	Charing Cross and Royal Marsden Hospitals, London, UK	Prospective	88 patients with early breast cancer T 0–2, N 0–1, M 0	Examination of the effect of psychological response to cancer disease on survival/recurrence 3 years post diagnosis and 5 years later
Reynolds *et al*. 1994 [15]	National Cancer Institute, Atlanta, Georgia; New Orleans, Louisiana; San Francisco, Oakland, California	Prospective	552 black and 486 white women with newly diagnosed breast cancer of any stage	Examination of the effect of social factors on breast cancer survival
Tross *et al*. 1996 [16]	Multi-centre study, USA	Prospective	280 women with stage II breast cancer who have undergone surgery and participated in a national clinical trial of CALGB involving 4 adjuvant therapy regimens	Examination of the effect of psychological factors on breast cancer survival/recurrence over a 15 year period
Buddeberg *et al*. 1996 [17]	Department of Gynaecology, Zurich and Basel, Switzerland, and Hospital Gynaecology and Obstetrics, Bad Sackingen, Germany	Prospective	107 women with early breast cancer who had undergone surgery 6 months earlier	Examination of the effect of different coping strategies on breast cancer survival, 5 to 6 years after primary surgical treatment
Kreitler *et al*. 1997 [18]	Five hospitals in the centre of Israel	Prospective	96 women with stage I, II breast cancer	Examination of the effect of psychological factors on breast cancer survival/recurrence
Tominaga *et al*. 1998 [19]	Tochigi Cancer Centre hospital, Saitama, Japan	Prospective	398 female patients who had undergone surgery for primary breast cancer between 1 September 1986 and 31 January 1995 at the Tochigi Cancer Centre Hospital and followed up in the outpatient clinic; some patients were treated with radiation and/or chemotherapy in addition to surgical treatment	Examination of the effect of social factors on breast cancer survival in a follow-up period up to the end of October 1995 or death
Brabander and Gerits 1999 [20]	The University Hospital of the Free University of Brussels, Belgium	Prospective	44 patients with stage I to III primary breast carcinoma (56 women were admitted to the University Hospital of the Free University of Brussels for a breast biopsy and 44 of them had a primary mammary carcinoma)	Examination of the effect of acute and chronic stress on breast cancer recurrence in 3.5 year follow-up period
Watson *et al*. 1999 [21]	Royal Marsden Hospital, London and Sutton, UK	Prospective	578 women with an early stage breast cancer (stages I and II), diagnosed 4 to 12 weeks before the inclusion date; their psychological response was measured 4 to 12 weeks and 12 months after diagnosis	Examination of the effect of psychological factors on breast cancer survival/recurrence in an at least 5 year follow-up period
Reynolds *et al*. 2000 [22]	National Cancer Institute, Atlanta, Georgia; New Orleans, Louisiana; San Francisco, Oakland, California	Prospective	442 black and 405 white women with invasive breast cancer of any stage diagnosed during 1985 to 1986	Examination of the effect of different coping strategies on breast cancer survival
Butow *et al*. 2000 [23]	Department of Medical Oncology, Sydney, Australia	Prospective	99 patients with metastatic breast cancer seen for the first time between 1991 and 1995 in the Department of Medical Oncology in a major teaching hospital in Sydney. Women entered the study a median of 4.7 months since diagnosis of distant metastases	Examination of the effect of psychosocial factors on breast cancer survival in a 2 year follow-up period
Weihs *et al*. 2000 [24]	Four academic medical centres in Washington, USA	Prospective cohort	32 (out of 65 eligible) patients with recurrent breast cancer diagnosed 6 to 19 months earlier and stabilized using surgical, medical and/or radiation therapies	Examination of the effect of psychological factors on breast cancer survival at 6 and 19 months after diagnosis
Graham *et al*. 2002 [25]	NHS Breast Clinic, London; 1991 to 1999	Prospective observational cohort	Consecutive series of 222 women diagnosed at Guy's Hospital between May 1991 and July 1994, aged 60 and under, newly diagnosed as having a primary operable breast tumour; 202/222 (91%) eligible women participated in the first life experiences interview, while 170 (77%) provided complete interview data either up to 5 years after diagnosis or recurrence	Examination of the effect of stress on breast cancer recurrence in a 5 year follow-up period
Soler-Vila *et al*. 2003 [26]	22 Connecticut hospitals, USA	Prospective cohort	145 African-American and 177 white women (out of 423 eligible women) diagnosed with a first primary breast cancer of any stage between January 1987 and March 1989	Examination of the effect of psychosocial factors on all-cause mortality and breast cancer mortality in 10 year follow-up period
Soler-Vila *et al*. 2005 [27]	22 Connecticut hospitals, USA	Prospective	145 African-American and 177 white women (out of 423 eligible women) diagnosed with a first primary breast cancer between January 1987 and March 1989	Examination of the effect of cancer-specific beliefs on breast cancer survival in a 15 year follow-up period
Hjerl *et al*. 2003 [28]	Denmark	Retrospective	20,589 women with breast cancer; 10,382 had early stage (tumour size <50 mm and no axillary lymph nodes infiltrated), 10,211 had late stage breast cancer (tumour size >50 mm, at least one infiltrated axillary lymph node, malignancy stage II or III in premenopausal women)	Examination of the effect of depression on breast cancer survival
Goodwin *et al*. 2004 [29]	Toronto, Ontario, Canada	Prospective	397 women diagnosed with breast cancer at participating University of Toronto teaching hospitals between October 1991 and May 1996. The patients were under 75 years of age, with T1 to T3, N0/N1, and M0 breast cancer; they represented women recruited during the final 5 years of a larger prospective cohort study examining prognostic effects of a number of lifestyle-related factors	Examination of the effect of health-related quality of life (HRQOL) and psychosocial factors on breast cancer survival/recurrence 2 months after diagnosis and 1 year later
Osborne *et al*. 2004 [30]	Four Melbourne public teaching hospitals; Preston and Northcote Community Hospital, Western Hospital, Maroondah Hospital, Dandenong Hospital	Prospective	62 women with first primary breast cancer who had no clinically obvious metastatic disease; women had completed chemotherapy treatment more than 4 weeks prior to their entry to the study	Examination of the effect of immune status and psychosocial factors on breast cancer survival in a 6.1 to 7.9 year follow-up period
Weihs *et al*. 2005 [31]	Washington, USA	Prospective	90 (out of the 183 eligible participants) women with invasive breast cancer stages II and III	Examination of the effect of social support on breast cancer survival in an 8 to 9 year follow-up period
Osborne *et al*. 2005 [32]	USA (the Surveillance, Epidemiology, and End Results (SEER) tumour registries is supported by the National Cancer Institute and includes registries in selected geographic areas: Detroit, San Francisco/Oakland, Atlanta, Seattle, Los Angeles and San Jose/Monterey, and the States of Connecticut, Iowa, New Mexico, Utah, and Hawaii)	Retrospective	32,268 women aged 65 years and older who were diagnosed with breast cancer of any stage from 1991 to 1995 and were followed over a 3 year period	Examination of the effect of marital status on breast cancer survival in a 3 year follow-up period
Lehto *et al*. 2006 [33]	Tampere University Hospital Oncology Clinic, Finland	Prospective	101 (out of 102) patients under 72 years of age with localized or regional breast cancer, who were admitted for treatment and/or follow-up to the Tampere University Hospital Oncology Clinic from January to October 1996	Examination of the effect of psychosocial factors on breast cancer survival
Kroenke *et al*. 2006 [34]	USA	Prospective study, based on the Nurses' Health Study (NHS) of 121,700 USA female nurses (at baseline in 1976, and biennially, participants provided health behaviour and medical history information through mailed questionnaires. A subset (*n *= 108,170) of these women also responded to social networks questions in 1992, 1996, or 2000	2,835 (out of 3,248) women aged 46 to 71 in 1992 from the Nurses' Health Study, who were diagnosed with stages 1 to 4 breast cancer between 1992 and 2002, without previous diagnosed cancer (except non-melanoma skin cancer) and who responded to social networks questions before diagnosis. Also, 1,753 women similarly diagnosed, without prior cancer, who completed social networks questions 1 to 4 years after diagnosis but before recurrence (defined as a second cancer on a routine NHS follow-up if lung, liver, bone, or brain cancer)	Examination of the effect of social factors on breast cancer survival in a 12 year follow-up period

**Table 2:** Methods and outcomes of studies examining the association of psychosocial factors and breast cancer survival

Reference	Method	Disease outcome	Psychological/psychosocial factors examined	Main findings
Greer *et al*. 1979 [4]	Clinical and psychological assessment of patients preoperatively and 3 and 12 months postoperatively and then annually for 4 years. Patients were interviewed and completed psychological tests. Rating scales were devised to measure social adjustment (marital, sexual, interpersonal relationships, work record). Depression was measured by Hamilton rating scale, hostility by Caine and Foulds hostility and direction of hostility questionnaire (HDHQ), extraversion and neuroticism by Eysnack personality inventory (EPI), intelligence by Mill Hill vocabulary scale. At follow up examination, ratings of social adjustment and of depression were repeated and patients' psychological responses to cancer were assessed	At 5 years follow up, 33 patients (49%) were alive and well without signs of recurrence, 16 patients (24%) were alive with metastases, 18 (27%) had died of breast cancer; 2 patients died of disorders other than cancer	Psychological responses: denial, fighting spirit, stoic acceptance, and helplessness/hopelessness. Psychosocial factors: social class, reaction on first discovering the breast lump, delay in seeking advice, reaction to stressful events, expression/suppression of anger, depression, hostility, depressive illness (during 5 previous years), psychological stress, sexual adjustment, interpersonal relationships, work record, extroversion, neuroticism, verbal intelligence	Recurrence-free survival was significantly more common among patients who reacted to cancer with denial or fighting spirit than among patients who responded with stoic acceptance, and helplessness/hopelessness
Derogatis *et al*. 1979 [5]	Initial evaluation of the patient at their second visit to outpatient department. Interview and assessment of psychological symptoms using the SCR-90 R, the Affect Balance Scale (ABS), the Global Adjustment to Illness Scale (GAIS), the Patients' Attitude, Information and Expectancy form (PAIE). The treating oncologist also completed GAIS and PAIE 3 days after the visit. Medical data were also recorded	13 patients lived less than 1 year and were characterized as short-term survivors; 22 patients lived more than 1 year and were characterized as long-term survivors	Joy, contentment, vigour, affection, anxiety, depression, hostility, psychoticism, guilt	Long-term survivors had higher psychological distress and showed higher levels of anxiety (*p *< 0.1), hostility (*p *< 0.01), psychoticism (*p *< 0.01), depression (*p *< 0.05), guilt (*p *< 0.05), negative affect total score (*p *< 0.01), positive symptom total (*p *< 0.1) and overall general severity index (*p *< 0.1)
Marshall *et al*. 1983 [6]	Each patient was asked about the occurrence of traumatic events 5 years before the first recognition of symptoms (deaths, illnesses, divorces, unemployment). Also patients were asked questions about the extension of social involvement (marital status, participation in religious/non-religious groups). Only women whose deaths have been recorded were included in the study	All patients died. Mean of survival of women <46 years in lower and higher stress quartile: 75.8 months and 50.8 months, respectively. Mean of survival of women 45 to 60 years in lower and higher stress quartile: 54 months and 39.2 months, respectively. Mean of survival of women >61 years in lower and higher stress quartile: 53.4 months and 54.9 months, respectively	Stress (deaths, illnesses, divorces, unemployment), social involvement (marital status, relatives and acquaintances, organization memberships)	The single most powerful predictor of survival is stage. The only statistically significant indicator is social involvement only for the younger age group
Jensen *et al*. 1987 [7]	Assessment of repressive defensiveness with Marlowe-Crown Social Desirability Scale, Bendig Short Form of the Taylor Manifest Anxiety Scale. Assessment of convergent validity with Sackeim and Gur's Self Deception and Other Deception Questionnaires. Assessment of attention to inner experience using Absorption Scale of Tellegen's Differential Personal questionnaire and Huba, Aneshensel, Singer's Short Form of the Imaginal Processes Inventory. The participants in the study also completed the Millon Behavioral Health Inventory. All three groups were equated on variables reflecting known or suspected genetic, hormonal and socioeconomic factors. The groups with cancer were also compared with regard to factors present at diagnosis associated with the progress of the disease at follow-up	At follow-up (G1, 336 days on average for the 11 patients who died and 636 days on average for the rest; G2, 734 days on average) four women in G1 were in remission, four women in G2 developed recurrence. One died from brain tumour unrelated to breast cancer	Repressive defensiveness, helplessness, expression of negative affect, chronic stress, positive constructive daydreaming	All five psychological variables were significant by *p *< 0.05. Repressive defensiveness, lower levels of expression of negative affect, higher levels of helplessness, chronic stress, positive constructive daydreaming were associated with poorer survival outcome
Jamison *et al*. 1987 [8]	Filling of eight questionnaires: Situation Response Scale of general trait anxiousness, General Well Being Scale, Health Value Rating Questionnaire, Self Esteem Inventory, Hostility Scale of the Multiple Affect Adjective Checklist, Zung Depression Scale, Spielberg Trait Anxiety Inventory, Multidimensional Health Locus of Control Scale. Demographic data were acquired at testing and medical data from charts	By the time of analysis all the patients had died from their illness. The patients were divided into two groups: short-term survivors (X = 10.8 months) and long-term survivors (X = 30.4 months)	Anxiety, adjustment, self-esteem, hostility, depression, health locus of control	No consistent differences found between short- and long-term survivors on any psychosocial variable assessed
Hislop *et al*. 1987 [9]	Patients were asked to complete short self-administered questionnaires (90% completed within 3 months of diagnosis). Information was obtained about types of usual activities, extroversion, neuroticism, self-esteem, locus of control, recent life events, coping behavior, psychiatric symptoms (including anxiety, anger, depression, cognitive disturbance such as forgetfulness, difficulty concentrating and making decisions). Clinical data were obtained from medical records	26 deaths (25 attributed to breast cancer) and 38 recurrences over the 4 years of follow-up	Types of usual activities, extroversion, neuroticism, self-esteem, locus of control, recent life events, coping behaviour, psychiatric symptoms (including anxiety, anger, depression, cognitive disturbance such as forgetfulness, difficulty concentrating and making decisions)	Involvement in expressive activities and low level of anger were associated with significantly better survival and disease-free survival. Extroversion was associated with significantly better survival. Low level of cognitive disturbance was associated with significantly longer disease free survival
Cassileth *et al*. 1988 [10]	Self-report questionnaire including items on seven factors; social ties and marital history, job satisfaction, use of psychotropic drugs, general life evaluation/satisfaction, subjective view of adult health, hopelessness/helplessness, (and one item found to predict survival in patients with cancer) perception of the amount of adjustment required to cope with a new diagnosis	Group II (only this group included breast cancer patients): the total sample of patients in this group was 158, since 3 patients were accrued to the study after the original analysis. Of that total, 7 (4.43%) died without recurrence of their malignancy, 1 (0.63%) was lost to follow-up and 1 (0.63%) developed a new primary breast cancer. Of the remaining 149 patients, 64 (43%) had documented recurrence of their disease, while 85 (57%) remained in remission	Social ties and marital history, job satisfaction, use of psychotropic drugs, general life evaluation/satisfaction, subjective view of adult health, hopelessness/helplessness, perception of the amount of adjustment required to cope with a new diagnosis	Group II: two psychosocial variables were significantly associated with time to recurrence. Those who scored in the middle third of the Hopelessness Scale had significantly lower risk of recurrence (RR = 0.52, 95% CI, 0.28 to 0.96; *p *= 0.028). Those who scored in the middle third of the total psychosocial score range had greater risk of recurrence (RR = 1.7, 95% CI 1.0 to 2.8; *p *= 0.04). In further analyses, using low and high groups as covariates and the middle group as reference, extent of disease was the only significant variable
Levy *et al*. 1988 [11]	Patients were assessed early in the course of hospitalization (before any treatment), reassessed 4 weeks later and at that time their physicians were asked to predict survival time. Patients were given a structured interview and filled out the Affect Balance Scale (ABS)	At the time of analysis 24 of 36 women had died of their disease. Average survival from date of recurrence was 2 years	ABS overall negative mood score, hostility score, overall positive mood score, joy score	Patients who expressed more joy in the baseline testing, who had longer disease-free survival, who were predicted by their physicians to live longer and had fewer metastatic sites had longer survival time (*p *< 0.001). Positive mood was also associated with living longer (*p *< 0.002), negative mood (*p *< 0.004) and hostility (*p *< 0.004) were associated with shorter survival
Ramirez *et al*. 1989 [12]	Adverse life events and life difficulties occurring during the postoperative disease free survival were recorded. Adverse life experiences were measured with Bedford College life events and difficulties schedule. Life events were rated as severe if they had threatening implications (that is, death of a husband or child). Difficulties that carried a pronounced threat and persisted at least 6 months were rated as severe (that is, taking care for a physically handicapped child)	The median interval free of disease for women who had a relapse was 30.5 months	Stressful events (severe, non severe) and difficulties (severe, non severe)	Relative risk for relapse: after severe events, 5.67 (1.57 to 37.20), *p *= 0.004; after severe difficulties, 4.75 (1.58 to 19.20), *p *= 0.004. Events of any severity or non-severe and difficulties of any severity or non-severe did not affect recurrence
Barraclough *et al*. 1992 [13]	Three home interviews at 4, 24, and 42 months after operation yielded data about the period from 1 year before surgery until 3.5 years afterwards. The Bedford college life events and difficulties schedule was used to assess life events. Interviews were taped and discussed by the investigators	Relapse of breast cancer was confirmed in 47 (23%) of the 204 breast cancer cases; 26 of these had died by the end of the follow-up period (4 to 42 months after operation); 1 patient died from a reason unrelated to cancer	Life events, social difficulties, depressive symptomatology	For severe life events or social difficulties during the year before surgery, the hazard ratio was 0.43 (95% CI 0.20 to 0.93); during the follow-up period, the hazard ratio was 0.88 (0.48 to 1.64). For prolonged major depression before surgery and during follow up, the hazard ratios were 1.26 (0.49 to 3.26) and 0.85 (0.41 to 1.79). respectively. For absence of a full confidant before surgery, figures were 0.93 (0.42 to 2.09), and during the follow-up period figures were 0.86 (0.38 to 1.93)
Morris *et al*. 1992 [14]	Interview at 3 months post diagnosis, further tests at 6 to 9 months post diagnosis and another interview at 12 months. Follow up at 5 years post diagnosis. Tests used: Wakefield Self Assessment Depression Inventory, Spielberger State Trait Anxiety Inventory, Multidimensional Health Locus of Control Scale, Courtauld Emotional Control Scale	At 5 year follow up: 20 patients had died, 8 were alive with recurrence, 79 were alive without evidence of recurrence	Patient's Responses to Diagnosis (PRD): fighting spirit (PRD 1), denial (PRD 2), anxious preoccupation (PRD 3), stoic acceptance (PRD 4), helplessness/hopelessness (PRD 5)	PRD code was associated with poorer survival *p *= 0.06. Association of PRD code with recurrence was not clear, *p *= 0.15. Patients with PRD 1 and 2 had a better prognosis than patients with PRD 3–5, though not statistically significant (*p *= 0.07 for overall survival and *p *= 0.19 for recurrence)
Reynolds *et al*. 1994 [15]	Information about two dimensions of social ties: structural, which reflect marital status, group participation, contacts with friends and relatives (the questions were based on Beckman SNI); and functional, which reflect perceived emotional support (the questions were based on Seeman's study of angiography patients). 87% of interviews took place within 6 months of diagnosis. Follow up information was obtained from Surveillance, Epidemiology, End Results database and National Cancer Institute	Data of survival reported in NCI Black White Cancer Survival Study	Marital situation, church group participation, other group participation, friends/relatives, contacts, social network index, emotional support, instrumental support	Absence of close ties and perceived sources of support were associated with increased breast cancer death rate. White women with few friends had increased death rate (RR = 2.1, CI 1.1 to 4.4). Black and white women with few sources of emotional support had increased breast cancer death rate (RR = 1.8, CI 1.3 to 2.5)
Tross *et al*. 1996 [16]	Assessment of psychological symptoms prior to chemotherapy using the Symptoms Checklist-90 revised questionnaire. Scores where trichotomized and Global Severity Index was extracted. Sociodemographic (age, ethnicity, education, and so on) and medical data were also collected	Disease free survival for patients with low, medium, high GSI was: 7.48, 7.04 and 4.90 years, respectively. Overall survival for patients with low, medium, high GSI was: 11.62, 10.21 and 9.58 years, respectively	Somatization, obsessive-compulsive symptoms, interpersonal sensitivity, depression, anxiety, hostility, phobic anxiety, paranoid ideation, psychoticism	This study failed to provide evidence that psychological factors contributed to length of disease-free or overall survival of women who received adjuvant chemotherapy for treatment of stage II breast cancer
Buddeberg *et al*. 1996 [17]	An interview every 12 months (sociodemographic data, attitude toward cancer and quality of life data) and 2 coping questionnaires: Zurich questionnaire of Coping with Illness (ZQCI) and Freiburg Questionnaire of Coping with Illness (FQCI). The ZQCI was administered every 3 months in the 1st year (assessments 1 to 5) and every 6 months during the 2nd and 3rd year (assessments 6 to 9). FQCI every 12 months (assessments 1, 5, 7, 9). ZQCI and FQCI are self-reported measures with five-point answer-scales for each item	5 to 6 years after the primary surgical treatment: 66.4% were recurrence-free and had no signs of cancer disease, 1.9% had a local-regional recurrence, 5.6% were afflicted with distant metastases, 23.4% had died from breast cancer, 1.9% developed a 2nd carcinoma, and 0.9% died from a cardiac disease	Coping strategies based on two questionnaires	There is no steady significant relationship between the different coping strategies and disease outcome
Kreitler *et al*. 1997 [18]	Filling of four questionnaires (background information, Psychosocial Adjustment to Illness Scale (PAIS), State-Trait Anxiety Inventory, Locus of control) by a mean of 13.34 months after surgery or 9.89 months after end of treatment. Medical parameters were also examined (stage of disease, estrogen/progesterone receptor status). The health state of patients was examined again 3 and 5 years after surgery	After 3 years: 73.96% were in a good state of health and 1.04% had died. After 5 years: 70.83% were in a good state of health and 14.58% had died	Adjustment, locus of control, anxiety	Both medical and psychological variables are significant predictors for good health state on 3 years and survival on 5 years. The most important psychological predictor was adjustment (especially with regard to sexual and social relations)
Tominaga *et al*. 1998 [19]	Examination of medical records and personal interviews with the patients at first admission	During the study period and up to October 1995, 48 (12%) patients died of breast cancer and 348 (87.4%) patients were alive	Marital status, having children, number of female and male children, participation in the meetings of patients, having a hobby, number of hobbies, religion, habit of smoking, and habit of drinking alcohol	Being a widow was significantly associated with decreased survival (hazard ratio 3.29, 95% CI 1.32 to 8.20, *p *= 0.011). Having a hobby was associated with longer survival (hazard ratio 0.43, 95% CI 0.23–0.82, *p *= 0.010). Number of hobbies was associated with longer survival (hazard ratio 0.64, 95% CI 0.41 to 1.00, *p *= 0.048). The presence of a child did not influence survival, but the number of female children of patients with breast cancer was associated with survival (hazard ratio 0.64, 95% CI 0.42 to 0.98, *p *= 0.039). Smoking was found to be a significant negative predictor of survival (hazard ratio 2.08, 95% CI 1.02 to 4.26, *p *= 0.044). Drinking alcohol had a positive impact on survival of breast cancer patients (hazard ratio 0.10, 95% CI 0.01 to 0.72, *p *= 0.023). Participation in the social circle of breast cancer patients and religion were not significant prognostic factors of survival
Brabander and Gerits 1999 [20]	Chronic stress was measured with the Dutch version of the Hopkins Symptom Check List on the day of admission. Acute stress was measured with the Dutch version of the Depression Adjective Checklist, before and after the communication of the biopsy results. Natural killer activity (NKA) and the number of NK cells (before and after the biopsy results) were determined as indicators of the immunological consequences of acute stress, while measurement of 3-methoxy-4-hydroxy-phenyl-glycol (MHPG; before and after the biopsy results) was used as a neurochemical indicator of acute stress. Patients were also interviewed by means of audiotaped semi-structured interviews on their expectation about the result of the biopsy	After a follow-up period of 3.5 years, 10 (22.7%) patients had relapsed; 9 of the 10 relapsed patients (90%) had developed metastases and 1 patient (10%) had developed a new carcinoma in the other breast	Acute stress and chronic stress	Acute stress and chronic stress were a significant predictor of early relapse at 3.5 years of follow-up
Watson *et al*. 1999 [21]	Participants completed self-administered questionnaires at 4 to 12 weeks after diagnosis and 1 year later. Mental Adjustment to Cancer (MAC) scale, Courtauld emotional control (CEC) scale, and hospital anxiety and depression (HAD) scale	At 5 years, 395 (68.3%) women were alive and without relapse, 50 (8.65%) were alive with relapse, and 133 (23%) had died (122 (21.1%) of breast cancer)	Patients' reactions to having cancer (fighting spirit, helplessness or hopelessness, anxious preoccupation, fatalism, and avoidance). The extent to which patients suppress negative emotions (anger, anxiety, sadness), and presence of depression or anxiety	Psychological assessment at baseline: women with a high score on the HAD scale category of depression had a significantly increased risk of death from all causes by 5 years (hazard ratio 3.59, 95% CI 1.39 to 9.24). Women with high scores on the helplessness and hopelessness category of the MAC scale had a significantly increased risk of relapse or death at 5 years, compared with women with a low score in this category (hazard ratio 1.55, 95% CI 1.07 to 2.25). Fighting spirit was not associated with disease outcomes. Psychological assessment at 1 year follow-up: the increased risk of death during follow up for the HAD scale category of depression remained significant (adjusted hazard ratio 4.04, 95% CI 1.54 to 10.64) for a score of >11 on HAD scale depression. The effect of MAC helplessness or hopelessness on event-free survival was reduced from baseline (hazard ratio 1.35, 95% CI 0.91 to 200) and was no longer significant
Reynolds *et al*. 2000 [22]	Patients were followed for survival through 1994 (since 1985 to 1986). They were administered a modified Folkman and Lazarous Ways of Coping Questionnaire. Coping strategies were characterized via factor analysis of the responses. Interviews took place within 3 months of diagnosis. Follow-up information was obtained from Surveillance, Epidemiology, End Results database and National Cancer Institute	218 deaths due to breast cancer (137 black women, 81 white). Median follow up for women who had not died: 107 months	Seven coping strategies; expressing emotions, wishful thinking, problem solving, positive reappraisal, avoidance, escapism	Expression of emotions was associated with better survival (hazard ratio 0.6, 95% CI 0.4 to 0.9). Women who reported both low expression of emotions and low perceived emotional support had shorter survival (hazard ratio 2.5, 95% CI 1.7 to 3.7)
Butow *et al*. 2000 [23]	Patients answered a series of questionnaires, repeated every 3 months for up to 2 years following study entry. In this paper there is only the analysis of the initial questionnaire of each patient. Self-report adaptation of the Weisman and Wordon General Coping Strategies Scale (COPE), Psychological Adjustment to Cancer scale (PAC), Multidimensional Support Scale (MDSS), Quality of Life measured by GLQ-8 LASA scales, and patients' perceptions of the aim of the treatment at the initial assessment only (four options: complete cure, increased long-term survival, increased short-term survival, or reduction of symptoms)	Survival was measured from date of study entry to date of death or censored at the date of last follow up for surviving patients; 63% died within the study period	Coping strategy, psychological adjustment, social support, quality of life, and perceptions regarding the aim of treatment	The adjustment style of minimization (minimizing the impact of cancer on social, work, and family life) was significantly associated with survival. Patients who minimized the impact of cancer survived longer (median of 29 versus 23.9 months after study entry (hazard ratio 0.932, CI 0.883 to 0.984, *p *< 0.01). The risk of dying was reduced by 7% for each unit increase in minimisation, and survival time was increased by a median of 5.2% months after study entry for those scoring above the median on minimisation
Weihs *et al*. 2000 [24]	The collection and interpretation of data regarding psychological assessments begun at least 6 months after recurrence and prior to the known terminal phase (that is after the acute adjustment to breast cancer recurrence and before the effects of terminal illness). Patients completed questionnaires; negative affectivity/mood was measured with the Profile of Mood States and the Taylor Manifest Anxiety Scale (MAS). Restriction of emotions was measured with the Control of Feelings Scale and the Marlowe Crowne (MC) Social Desirability Scale. Chronic anxiety and emotional constraint scores were used to create four emotion regulatory style groups: low anxiety/low emotional constraint, low anxiety/high emotional constraint, high anxiety/low emotional constraint, and high anxiety/high emotional constraint	At end of data collection, 19 of 32 patients had died. Median time from first occurrence to death or last observation was 2.5 years (range 0.9 to 4.4 years). Median survival time after study enrolment to death of last observation was 1.7 years (range 02 to 3.2 years)	Negative affectivity, negative mood states, and restriction of emotions	Bivariable analysis: low chronic anxiety in the context of low emotional constraint predicted low mortality (RR = 0.07, CI 0.009 to 0.52, *p *= 0.01; low chronic anxiety scores but with high emotional constraint had higher mortality (RR = 3.73, CI 1.21 to 11.51, *p *= 0.02); high chronic anxiety was associated with increased mortality risk only after the patient's level of emotional constraint was taken into account; emotional constraint, regardless of chronic anxiety level (RR = 6.45, CI 2.17 to 19.16, *p *= 0.001), and controlled feelings (RR = 3.41, CI 1.0 to 12.0, *p *= 0.05) predicted increased mortality; negative mood states predicted mortality (RR = 2.64, CI 1.36 to 5.12, *p *= 0.004), but chronic anxiety when analyzed separately did not do so. Multivariable analysis: Control of feelings remained a significant predictor of survival independent of the dichotomized variable low anxiety/low emotional constraint group versus others (RR = 5.69)
Graham *et al*. 2002 [25]	Collected data on stressful experiences and depression, interviews (tape recorded and transcribed) carried out every 18 months and covered the period from 12 months before diagnosis to 5 years after diagnosis. Final interview with patients who had a recurrence took place approximately 8 weeks after diagnosis. Collected data on stressful life experiences by using the Bedford College life events and difficulties schedule. At each interview elicited psychiatric symptoms using the structured clinical interview with criteria from the DSM III R	Recurrence of disease was confirmed in 54 (31.6%) women. The overall five year relapse-free survival was 76% (95% confidence interval 68.6 to 81.1) Some patients died soon after recurrence, and four, out of the final interviews, were completed by the woman's closest relative	Discrete life events and longstanding difficulties (for example, divorce, caring for a severely handicapped child)	No evidence that women who have a severely stressful life experience in the year before breast cancer diagnosis, or in the 5 years following are at any increased risk of developing a recurrence of their disease. An episode of depression before diagnosis did not increase the risk of recurrence (hazard ratio 1.22, CI 0.38 to 3.92, *p *= 0.7). No increased risk of recurrence in women who had had one or more severely stressful experiences in the year before diagnosis compared with women who did not (hazard ratio 1.01, CI 0.58 to 1.74, *p *= 0.99). In the post-diagnosis period, women who had one or more severely stressful life experiences had a lower risk or recurrence than those who did not (hazard ratio 0.52, CI 0.29 to 0.95, *p *= 0.03)
Soler-Vila *et al*. 2003 [26]	42% of the patients were interviewed within 3 months, 90% within 6 months of the date of diagnosis. Participants were interviewed in their homes with the use of a modified version of the questionnaire used in the National Cancer Institute Black/White Cancer Survival Study; this instrument collected information on sociodemographic, health history, medical care, and psychosocial factors	Survival was defined as time from breast cancer diagnosis until death or censoring by last contact date. During the study period there were 135 (41.9%) deaths from any cause and 99 (30.7%) deaths from breast cancer	Coping styles (denial, passive coping, and isolation), perceived emotional support, fatalism, and health locus of control	Lower perceived emotional support at diagnosis was associated with a higher risk of death from any cause (hazard ratio 1.39, 95% CI 1.09 to 1.79, *p *= 0.009) as well as death from breast cancer (hazard ratio 1.43, 95% CI 1.07 to 1.92)
Soler-Vila *et al*. 2005 [27]	42% of the patients were interviewed within 3 months, 90% within 6 months and the remaining 10% within 1 year of the date of the diagnosis. Interviews were based on a modified version of the questionnaire used in the National Cancer Institute Black/White Cancer Survival Study, which gathered information on sociodemographics, health history, medical care, and psychological factors	Survival was defined as time from breast cancer diagnosis until death or censoring by last contact date. By the end of the study period (2002), there were 160 deaths from any cause (49.7%), out of which 105 were due to breast cancer (65.6%), 55 due to diseases of the circulatory system (13%) and 13 due to other neoplasms (8%)	Beliefs about cancer detection, treatment, and curability of cancer disease	Perceived cancer incurability was associated with a higher risk of death from any cause (all-cause mortality; hazard ratio 1.67, 95% CI 1.11 to 2.51), though not associated with breast-cancer mortality
Hjerl *et al*. 2003 [28]	Information about depression was obtained from the Danish Psychiatric case register, which provides information for admissions to all psychiatric departments and hospitals in Denmark. Records of the patients were obtained from Danish Breast Cancer Cooperation Group. Information about death obtained from Mortality Danish National Register of causes of death	During the study period (1977 to 1993) 5,648 patients (27.4%) died from natural causes and 79 patients (0.4%) died from unnatural causes	Depression defined as all affective and anxiety disorders divided and categorized into five ordinal groups (bipolar, unipolar, reactive disorder, dysthymia, anxiety). Preoperative depression was defined as any psychiatric admission with any of the above mentioned ordinal groups at >15 years of age, from April 1969 to December 1993, at least 3 months before breast cancer diagnosis	Preoperative depression was associated with significantly higher relative risk of mortality for late stage breast cancer. The same trend but non-significant was seen in the early stage breast cancer group. The most pronounced effect was noticed in patients with bipolar diagnosis (compared with the other diagnosis)
Goodwin *et al*. 2004 [29]	Completion of health-related quality of life (HRQOL) and psychosocial questionnaires shortly after breast cancer diagnosis and 1 year later (sent and returned by mail). European Organization for Research and Treatment of Cancer Quality of Life Questionnaire-Core 30 (EORTC QLQ-C30), Profile of Mood States (PMOS) questionnaire, Impact of Events Scale (IES), Mental Adjustment to Cancer Scale (MAC), Courtauld Emotional Control Scale (SECS), Psychosocial Adjustment to Illness Scale Self-Report (PAIS-SR). Women were followed through an ongoing review of medical records	50% of the patients were followed ≥6 years, 3 (0.75%) women were followed up for less than 1 year, 55 (13.85%) experienced distant recurrences, and 34 (8.56%) women died of breast cancer; 2 (0.5%) women experienced non-breast cancer-related deaths	Health-related quality of life, mood state, stress response syndrome, coping strategies, emotional control, psychological adjustment to illness	No consistency of associations across outcomes or questionnaires between health-related quality of life and psychosocial status and breast cancer prognosis. There was little evidence of significant prognostic associations of 1 year psychosocial measurements. Out of 140 investigated prognostic associations, only 4 were statistically significant: role functioning (EORTC QLQ-C30) was significantly associated with overall survival (OS; *p *= 0.031), suggesting that better functioning was associated with a lower risk of death. Cognitive functioning (EORTC QLQ-C30) was associated with distant disease-free survival (DDFS; *p *= 0.041), suggesting that women who had better cognitive functioning 1 year after breast cancer diagnosis had a reduced DDFS. On the PAIS, domestic environment was associated with OS (*p *= 0.049), suggesting that women who experienced a great deal of illness and disease on domestic environment had an increased risk of death. The avoidance subscale on the IES was associated with OS (*p *= 0.014), suggesting that women who scored higher on this subscale had a significant lower risk of death
Osborne *et al*. 2004 [30]	Hospital Anxiety and Depression Scale (HADS), Duke-UNC Functional Social Support (DUFS), Mental Adjustment to Cancer (MAC). Approximately two-thirds of the patients were interviewed between 5 and 9 months following diagnosis, the remaining one-third was interviewed 9 to 17 months following diagnosis	Minimum time from interview to follow-up was 6.1 years and maximum 7.9 years. Survival was measured from date of interview to date of death from breast cancer, or was censored at the date of last follow-up or date of death from other causes; 18 (29%) patients had died, 14 (23%) from breast cancer, 3 (4.8%) from other causes, and in 1 case the cause of death could not be verified; 5 years after diagnosis there were 11 deaths (18%)	Anxiety, depression, mental adjustment to cancer and social support	Fighting-spirit minimizing the illness; high score in MAC subscale was associated with longer survival (HR 0.77, *p *= 0.008). No statistically significant effects of anxiety, depression or social support on survival were found. Leukocyte numbers were found to be weakly associated with helplessness and depression (r = 0.26 and 0.29, respectively), T-suppressor cell number with fatalism (0.29), and the lymphocyte stimulation index with confidant support (-0.30) and depression (0.25). Cortisol and prolactin levels were not found to be significantly associated with immune measures or psychosocial measures. No substantial evidence for a psychoneuroimmunological link between psychosocial factors and biological factors.
Weihs *et al*. 2005 [31]	The number of dependable support persons and the amount of contact with them were assessed within 18 months after diagnosis in women with stage II or III breast cancer. At the time of enrolment (1991 to 1993) patients were assessed for support and disease severity (with the use of the Nottingham Prognostic Index (NPI)) and then monitored for disease status through December 2000. Participants listed the first names of supportive relatives and friends and gave the frequency of contact with each person. To estimate the disease outcome, participants' medical charts were reviewed annually. Treatment aggressiveness was also assessed	Time to recurrence or death was set at the date of enrolment to the study (1991 to 1993) until December 2000. At the end of the study period there were 16 (17.7%) deaths from breast cancer and 21 (23.3%) recurrences, while 3 (3.33%) participants died from other causes	Social support: the number of supportive relatives and friends and the frequency of contact with each one	Survival analyses using the number of dependable supports and NPI (disease severity) as simultaneous predictors showed decreased mortality in participants with a 1 standard deviation increase in dependable supports (SD = four dependable supports): (RR = 0.41, CI 0.21 to 0.80, *p *= 0.01), and a trend toward decreased recurrence (RR = 0.68, CI 0.42 to 1.1, *p *= 0.11)
Osborne *et al*. 2005 [32]	Data were from the Surveillance, Epidemiology and End Results (SEER) tumour registries, merged with Medicare data for the years 1991 through 1995. From the SEER program, information regarding tumour location, size, axillary node status, American Joint Committee on Cancer Stage (AJCC), estrogen receptor status, demographic characteristics (age, sex, race, and marital status), and types of treatment provided within 4 months after the date of diagnosis was obtained. The data from the Medicare program used in the study were the Medicare Provider Analysis Review File, the Hospital Outpatient Standard Analytic file, and the 100% physician/supplier file. Information available through 1998 allowed for 3 years follow-up		Marital status	Unmarried women were at an increased risk of death from breast cancer (hazards ratio 1.25, 95% CI 1.14 to 1.37)
Lehto *et al*. 2006 [33]	Interviews of the patients based on the Ways of Coping questionnaire (WOC), the MOS Social Support Survey, the Anger Expression Scale (AX/Scale), Life Experience Survey (LES), the Rotterdam Symptom Checklist (RSCL), and the Depression Scale (DEPS)	Survival was measured from the day of diagnosis to the day of advancement of the disease (event-free survival) or date of death (overall survival) or day of the last follow-up (15 February 2005). At 15 February 31 patients had relapsed (30.7%) and 20 (19.8%) had died	Coping strategies, emotional expression, anger expression, perceived available social support, non-cancer life stresses, and quality of life 3 to 4 months after diagnosis	In univariate analysis: emotional defensiveness (*p *= 0.007) and behavioural escape-avoidance (*p *= 0.057) were significantly associated with shorter survival. In multivariate analysis: distancing (minimising) coping was associated with longer survival (*p *= 0.034). Emotional defensiveness (anti-emotionality; *p *= 0.021), behavioural escape-avoidance coping (*p *= 0.008), and high level of perceived support (*p *= 0.009) were associated with shorter survival. Depressive symptoms had a survival-decreasing effect before the coping patterns were added into the final models (*p *< 0.050). Depressive symptoms and high-perceived support tended to predict a shorter event-free time survival (time without relapse; *p *= 0.066 and *p *= 0.074, respectively).
Kroenke *et al*. 2006 [34]	Social networks were assessed in 1992, 1996, and 2000 with the Berkman-Syme Social Networks Index (SNI). Social support was assessed in 1992 and 2000 as the presence and availability of confidant. Social-emotional support was assessed in 1992 and 2000 as the presence and availability of a confidant	224 deaths (107 of these related to breast cancer) accrued to the year 2004	Social-emotional support (presence and availability of a confidant and the frequency of communication with him/her) and social networks (marital status, church group membership, membership in other community organizations)	Prediagnostic analyses of social networks and survival: women who were socially isolated before diagnosis had a subsequent 66% increased risk of all-cause mortality (hazards ratio (HR) = 1.66; 95% CI 1.04 to 2.65) and a two-fold increased risk of breast cancer mortality (HR = 2.14, 95% CI 1.11 to 4.12) compared with those who were socially integrated. The presence and extent of contact with a confidant was associated with survival. Women without close relatives (HR = 2.65, 95% CI 1.03 to 6.82), friends (HR = 4.06, 95% CI 1.40 to 11.75), or living children (HR = 5.62, 95% CI 1.20 to 26.46) had increased risk of breast cancer mortality and of all-cause mortality (HR = 1.66, 95% CI 0.93 to 2.97; HR = 2.20, 95% CI 1.01 to 4.81; and HR = 4.03, 95% CI 1.65 to 9.86, respectively) after diagnosis, compared with those who were socially integrated. Participation in religious or community activities, having a confidant and being married were not associated with survival. Postdiagnostic analyses of social networks and survival: lower social networks, following diagnosis, were not significantly associated with survival. Participation in group activities postdiagnosis appeared to predict a slightly lower risk of mortality (HR = 0.70, 95% CI, 0.44 to 1.11). The presence and extent of contact with a confidant was not associated with survival

**Table 3:** Characteristics of studies examining the effect of psychological intervention on breast cancer survival

Reference	Setting	Type of study	Study population	Aim of the study
Spiegel *et al*. 1989 [35]	California, USA	Prospective	86 women with metastatic carcinoma of the breast. Intervention group: 50 patients. Control group: 36 patients	Examination of the effect of psychosocial intervention on survival of patients with metastatic breast cancer
Gellert *et al*. 1993 [36]	New Haven, Connecticut, USA	Retrospective cohort study	102 non-participants and 34 participants in the psychological support program were matched on major prognostic factors. Both groups were monitored from the day of cancer diagnosis (1971 through 1980) until March 1991	Examination of the effect of a psychosocial intervention on breast cancer survival
Cunningham *et al*. 1998 [37]	Princess Margaret Hospital, Toronto, Canada	Prospective case control study	Intervention group: 30 patients with metastatic breast cancer. Control group: 36 patients with metastatic breast cancer	Examination of the effect of a psychological intervention on breast cancer survival
Edelman *et al*. 1999 [38]	Australia	Prospective case control study	121 patients with metastatic breast cancer, of whom 60 participated in the cognitive behavioural therapy program (therapy group) and 61 did not (control group	Examination of the effect of a psychological intervention on breast cancer survival
Shrock *et al*. 1999 [39]	Two rural hospitals or cancer centres in Pennsylvania	Matched case control	Intervention group: 21 patients with stage I breast cancer. Control group: 74 patients with stage I breast cancer	Examination of the effect of a psychological intervention on breast cancer survival
Goodwin *et al*. 2001 [40]	Samuel Lunenfeld Research Institute at Mount Sinai Hospital, University of Toronto. Also participated: Hamilton Regional Cancer Center, Hamilton, Ontario; Ottawa Regional Cancer Center, Ottawa Ontario; Cancer Care, Manitoba, Winnipeg; Tom Baker Cancer Center, Calgary, Alberta; British Columbia Cancer Agency, Vancouver; Cross Cancer Institute, Edmonton, Alberta	Prospective	235 women with metastatic breast cancer, expected to survive at least 3 months (intervention group, 158 women; control group, 77 women)	Examination of the effect of a psychological intervention on breast cancer survival

**Table 4:** Methods and outcomes of studies examining the effect of psychological intervention on breast cancer survival

Reference	Method	Disease outcome	Psychological/psychosocial factors examined	Main findings
Spiegel *et al*. 1989 [35]	Patients were randomly assigned to the intervention or the control group, by a mean of 24.4 months from first metastasis (intervention group) and 21.9 months from first metastasis (control group). The 1 year intervention consisted of weekly supportive group therapy, led by a psychiatrist or social worker with a therapist who had breast cancer in remission. Groups were encouraged to discuss how to cope with cancer, express their feelings, be more assertive with doctors. They were not led to believe that they would live longer. Self hypnosis was taught for pain control	At 10 years follow up only 3 patients were alive. Survival for the intervention group was a mean of 36.6 months and for the control group survival was a mean of 18.9 months	Participation in a psychosocial support therapy program	Patients in the intervention group lived significantly longer (*p *< 0.0001, Cox). The interval from first metastasis to death was also significantly longer (*p *< 0.001, Cox).
Gellert *et al*. 1993 [36]	Support program (Exceptional Cancer Patients (EcaP)) consisted of weekly cancer peer support and family therapy, individual counselling, and use of positive mental imagery, meditation, and relaxation. Using data from local hospital tumour registries in 1981, three women with breast cancer were matched to each EcaP participant regarding age at histological diagnosis, race, stage of disease, surgery, and sequence of malignancy. Subjects retrospectively monitored for survival time through March 1991, using records from the State of Connecticut Tumor Registry. Two analyses were made, an unadjusted and a restricted one. In the latter, non-participants were restricted to those who had a survival time greater than that of the matched case at time of entry into the support program	Unadjusted analysis: 41% of EcaP participants survived 10 years, compared with 47% of non-participants. Restricted analysis: 37% of non-participants survived 10 years	Participation in a psychosocial support therapy program	There was no significant association between psychosocial intervention and breast cancer survival
Cunningham *et al*. 1998 [37]	The intervention group was offered 35 2-hour weekly sessions of supportive and cognitive behavioural therapy. Patients in the intervention group were also given 20 cognitive behavioural assignments as homework and were encouraged to attend a weekend training in coping skills, especially relaxation, stress management, thought monitoring, goal setting and mental imaging. The control group received only a home study cognitive behavioural package	At 5 years follow up median survival on the intervention group was 28.2 months. Median survival on the control group was 23.6 months (probably due to the longer medial interval between metastatic diagnosis and entering the study in the control group)	Participation in supportive plus cognitive behavioural therapy groups	There was no significant association between psychosocial intervention and breast cancer survival
Edelman *et al*. 1999 [38]	Initially, 124 patients were recruited for the study, of whom 62 were randomly allocated to the intervention group and received cognitive behaviour therapy, whereas 62 were allocated to a standard care control group. Three patients (two from the therapy group and one from the control group) were excluded because they did not have metastatic disease. Patients were recruited between March 1994 and February 1997; thus, at the time of survival analysis participants had entered the study between 2 and 5 years previously. Of these patients, 38 had been identified through the medical records of the Royal North Shore Hospital, Sydney; doctors at other hospitals referred 3 and 83 responded to media publicity. Demographic information from patients, medical information from hospital records. Treating physicians completed Eastern Cooperative Oncology Group (ECOG) Performance status and Disease Status forms at each assessment period. Two self-report instruments for information on psychological criteria: the Profile of Mood States and the Coopersmith Self-esteem Inventory. Assessments were conducted prior to intervention, following intervention, and at 3 and 6 months after intervention.	Survival time was calculated from the day of entry to the study. At the time of survival analysis, 85 (70.2%) patients had died	Participation in a group cognitive behavioural therapy program	There was no significant association between psychosocial intervention and breast cancer survival
Shrock *et al*. 1999 [39]	Six two-hour psychology classes. Class topics: beliefs/feelings/attitudes toward health, relaxation/imagery techniques, nutrition, exercise, stress management, self-esteem, spirituality, receptive imagery/intuition, problem solving and personal health plan setting. The 21 breast cancer patients who received intervention were matched with 74 breast cancer patients of the same stage. There were two to three matched controls for each patient	At 4 to 7 years follow up none of the intervention group had died, 12% of the control group had died (median follow-up 4.2 years)	Participation in health psychology classes	The intervention control group lived significantly longer (*p *= 0.042)
Goodwin *et al*. 2001 [40]	Women participating in the intervention group attended weekly meetings lasting 90 minutes. Therapy intended to foster support among members and encourage the expression of emotions about cancer and its effects on their lives. Patients were encouraged to attend sessions for at least 1 year. The primary outcome was survival and the secondary psychosocial function assessed by self-report questionnaires (Profile of Mood States and Pain Suffering Scales by Spiegel-Bloom) at baseline and at 4, 8 and 12 months	By the time of analysis 201 patients had died. The median survival for the intervention group was 17.9 months, and for the control group was 17.6 months	Participation in supportive-expressive group therapy	There was no significant association between psychosocial intervention and breast cancer survival

**Table 5:** Association of the examined psychosocial factors with survival/recurrence

	Survival	Recurrence	No impact
Coping			[9], [17], [23], [26]
Cognitive/role functioning	+ [29]	+ [29]^a^	
Beliefs about cancer incurability			[27]
Fighting spirit	+ [30]	-[4]	[14], [21], [29]
Stressful events	-[29]	+ [12], -[13], -[25]^b^	[6], [9], [13]^c^, [25]
Anxiety	-[7]	+ [20]	[4], [5], [8], [9], [14], [16], [21], [29], [30]
Hopelessness/helplessness	-[7]	+ [4], + [10], + [21]	[14], [21]^d^, [29], [30]
Joy	+ [11]		[5]
Depression/negative mood	+ [5], -[11], -[21], -[24], -[28], -[33]		[4], [8], [9], [13], [16], [25], [30], [29], [33]^e^
Perceived social support	+ [15], + [26], -[33]		[30], [33]^f^
Social support	+ [6]^g^, + [15]^h^, + [31], + [34]		[4], [10], [13], [23], [30], [34]^i^
Minimizing	+ [23], + [33]		
Repressive defensiveness/emotional constraints	-[7], -[24], -[33]		[4], [21], [33]^j^
Adjustment	+ [18]^k^		[4], [8]
Fatalism/stoic acceptance		+ [4]	[14], [21], [26], [29], [30]
Positive constructing daydreaming	-[7]		
Denial/avoidance	+ [29], -[33]	-[4]	[14], [21]
Locus of control			[8], [9], [18], [26], [30]
Anger/hostility	+ [5], -[9], -[11]	+ [9]	[4], [8], [16], [29]
Vigor/activity			[5], [29]
Fatigue/inertia			[29]
Guilt	+ [5]		
Extroversion	+ [9]		[4]
Expressive activities	+ [9]	-[9]	
Group participation (religious/non-religious)	+ [6]		[15], [19], [34]
Confusion/bewilderment			[29]
Somatization, obsessive-compulsive symptoms, interpersonal sensitivity, paranoid ideation, psychoticism	+ [5]		[4], [9], [16]
Self-esteem			[8], [9]
Hobbies	+ [19]		
Female child	+ [19]		
Marriage	+ [6], + [19], + [32]		[10], [15], [29], [34]

The number in brackets is the number of the reference. Symbols: +, increased survival/recurrence; -, decreased survival/recurrence. ^a^Cognitive functioning. ^b^Post-diagnosis. ^c^During follow-up period. ^d^At 1 year follow up. ^e^Recurrence. ^f^No impact on recurrence. ^g^Younger age groups. ^h^White women. ^i^Post-diagnosis. ^j^Recurrence. ^k^Sexual adjustment. Minimisation: elimination of the impact of cancer on social, work, and family life. Perceived emotional support: reflects participant's ability to talk about disease related distresses with family/friends. Locus of control: reflects patient's beliefs about whether health outcomes are under control of powerful others/random forces. Emotional defensiveness: suppression and control of emotions.

Out of the 31 studies, 25 (80.6%) revealed that various psychological factors are significantly associated with survival/recurrence. Increased survival is associated with role functioning (one study [29]), fighting spirit (one study [30]), joy (one study [11]), depression (one study [5]), perceived social support (two studies [15,26]), social support (four studies [5,15,31,34]), minimization (two studies [23,33]), adjustment (one study [18]), denial (one study [29]), anger (one study [5]), guilt (one study [5]), extroversion (one study [9]), expressive activities (one study [9]), participation in religious/nonreligious groups (one study [6]), psychiatric symptoms (one study [5]), hobbies (one study [19]), female child (one study [19]), and marriage (three studies [6,19,32]). Decreased survival is associated with stressful events (one study [29]), anxiety/stress (one study [7]), hopelessness (one study [7]), higher perceived emotional support (one study [33]), depression (five studies [11,21,24,28,33]), repressive defensiveness (three studies [7,24,33]), positive constructive daydreaming (one study [7]), denial/avoidance (one study [33]). Cognitive functioning (one study [29]), stressful events (one study [12]), anxiety (one study [20]), hopelessness (three studies [4,10,21]), fatalism (one study [4]) and anger/hostility (one study [9]) were associated with higher recurrence rates. Fighting spirit (one study [4]), stressful events (two studies [13,25]), denial (one study [4]) and expressive activities (one study [9]) significantly reduced recurrence rates.

As seen in Table 5, for most of the separate psychosocial factors examined with regard to breast cancer survival and/or recurrence there was only one study supporting the association (either a positive or a negative impact on survival/recurrence). For several psychosocial factors (specifically, depression, social support, marriage, perceived support, stressful events, denial/avoidance, minimizing, repressive defensiveness, anger/hostility and hopelessness/helplessness) more than one study reporting a statistically significant association with breast cancer survival. For five of these eight psychosocial factors (stressful events, depression, perceived social support, anger/hostility, denial/avoidance) there are contradictory results (showing both negative and positive effects) on survival/recurrence. For example, while five studies show that depression is associated with decreased survival, there is one study where depression seems to increase survival. Perceived social support, social support, minimization and marriage were associated with prolonged survival in more than one study. Similarly, repressive defensiveness and depression are associated with decreased survival in more than one study. As far as recurrence is concerned, only stressful events appeared to reduce recurrence rates while hopelessness appeared to increase recurrence rates in more than one study.

In the 6 studies that used psychological intervention, a total of 389 female patients with breast cancer were examined (34 of them in a retrospective study). Psychological intervention varied from study to study, including group therapy, behavioral/cognitive therapy, peer support/family therapy, counseling, mental imagery, meditation, and psychology classes. Three of these studies were conducted in the US [35,36,39], two in Canada [37,40] and one in Australia [38]. Four studies examined patients with metastatic breast cancer, one patients with early breast cancer and one patients with breast cancer of any stage. Two studies showed that there is a survival benefit for those patients that received psychological intevention [35,39], while in four studies there was no influence on survival [36-38,40].

## Discussion

From the results of our review of the literature there are conflicting data regarding the possible association of psychosocial factors and survival. Various methodological issues of the examined studies have to be taken into account to interpret their results. Hence, while most of the examined studies in our review are prospective, the majority of the patients studied were in the retrospective studies. This means that the sample size of most of the prospective studies is relatively small. The follow-up period of the reviewed studies also differs significantly. One must be cautious when drawing conclusions from a variety of studies that used different assessment tools to evaluate the influence of psychosocial factors on the survival of patients with breast cancer. For example, in the study by Goodwin and colleagues [29] a psychosocial parameter (avoidance) was found to be important when assessed with a particular questionnaire, but this finding was not substantiated when using another questionnaire. Moreover, in the same study, the number of statistically significant associations of psychosocial factors with breast cancer survival actually found was less than that expected by chance.

Despite these limitations, some useful data regarding the role of psychosocial factors in breast cancer survival become evident. There is an overlap between studies that showed statistically significant associations and those that did not. This means that while a study may have reported a statistically significant association for a psychosocial parameter, it did not show significant correlations for other parameters. The small patient numbers in these studies may have precluded statistical significance. In the majority of the studies that showed a statistically significant association between a psychosocial factor and survival/recurrence, this association was either positive (increased survival/recurrence) or negative. For only four of the studied psychosocial parameters (depression, perceived social support, anger/hostility, denial/avoidance) were both positive and negative results evident. Thus, with regarding to depression, four studies showed increased patient mortality, five studies showed no effect at all and one study showed a contradictory effect, that is, better survival.

Parameters associated with better breast cancer prognosis are social support, marriage, minimizing and denial. Data from the literature regarding the effect of these factors on the survival of other types of cancer vary. Thus, in a recent systematic review no statistically significant influence of psychological coping on cancer survival (various types of cancer included) was found [41]. On the contrary, data from patients with melanoma show that married patients and patients adjusting with minimization also survived longer [42]. In our review, depression and constraint of emotions are associated with decreased breast cancer survival; the role of these factors has not been verified in all studies. In a prospective study of patients with various types of cancer, depression was found to be associated with cancer prognosis after 30 months of follow-up [43].

Other studies included in our review have shown limited and/or contradictory data for parameters such as fighting spirit, having a female child, showing adjustment, having hobbies, coping, role functioning, stressful events, stoic acceptance, fatalism, locus of control and anger.

Understanding the definitions of these psychosocial parameters is crucial. Closely related terms are not synonymous. Thus, one has to differentiate denial and minimizing (associated with better survival) from constraint of emotions (associated with shorter survival). Denial refers to the belief that one has no disease, while minimization refers to minimizing the seriousness of the disease. The constraint of emotions is not synonymous with denial/minimization. One may constrain his/her emotions while not denying the disease.

One cannot emphasize the need for repeated evaluations of the psychosocial factor over a protracted time period. This holds true as these factors may change over time, that is, they are dynamic in nature. Chronic versus acute changes in the psychosocial factors were not a particular focus in the reviewed studies, apart from one. Neither was any specific mention of the histological type of breast cancer made in these studies. In contrast, the majority of the studies reported on breast cancer stage; it seems that no association between psychosocial factors and the stage of the neoplasm (metastatic versus non-metastatic) was evident.

When examining the effects of various psychosocial factors on the outcome of patients with breast cancer, other possibly interacting factors (either psychosocial or biological) may be deemed to be confounders. It should be kept in mind that when excluding these factors that are potentially in interplay with the examined parameter from the analysis, the effect of the psychosocial factor might not become evident. For example, in breast cancer patients with a hormone (estrogen and/or progesterone) receptor positive status (biological factor), life events were related to recurrence of breast cancer, while such a relationship did not occur in women with hormone receptor negative breast cancer [12]. In an analogy, the effect of one psychosocial parameter may only become evident when examined in the context of other interacting psychosocial parameters. An example is shown in the study by Weihs and colleagues [24]; the effect of chronic anxiety varies when various levels of constraints of emotion are examined. Other studies do not confirm the interplay of psychosocial and biological factors [16].

The effect of various psychological interventions on the survival of breast cancer was examined in six studies and two of them showed a positive result. While psychological interventions improve the quality of life of patients with breast cancer, [35] their effect on actual survival is less obvious. Thus, it seems that psychological interventions have minimal effect on the prognosis of breast cancer patients. One has to acknowledge that the numbers of patients in the studies using psychological intervention are too small to reach definite conclusions. Data from the literature regarding the effect of psychosocial interventions on cancer survival are scarce; even data on the effects of these interventions on quality of life are contradictory [44-46].

## Conclusion

The majority of studies show a significant relationship between psychosocial factors and survival, but the actual psychosocial variables related to survival are not consistently measured across studies and the findings for many of the psychosocial variables with survival/recurrence are not consistent across studies. In particular, more research is probably warranted regarding the role of social support, marriage, minimizing, denial, depression and constraint of emotions on breast cancer survival. Adequately powered multicentre studies, the use of a few valid assessment tools and meta-analytical approaches may be necessary to show the potential roles of various psychosocial factors in breast cancer outcome.

## Abbreviations

CI = confidence interval.

## Competing interests

The authors declare that they have no competing interests.

## Authors' contributions

MEF had the original idea for this article. The other authors collected the relevant data. All authors contributed in the interpretation of the data. EAZ, EI, and PIR wrote different parts of the first version of the manuscript and MEF revised it for important intellectual content. All authors participated in subsequent revisions of the manuscript and approved its final version.
